# Trends in Japanese Spotted Fever (JSF) Outbreaks: Situation in the Eastern Region of Kochi Prefecture in Japan, a High-incidence Area

**DOI:** 10.31662/jmaj.2022-0131

**Published:** 2022-09-26

**Authors:** Ichiro Fukunaga

**Affiliations:** 1 Aki Public Health and Welfare Office, Kochi Prefectural Government, Kochi, Japan

**Keywords:** Japanese spotted fever, eschar, rash, eastern region of Kochi Prefecture, surveillance

## Introduction

**Figure 1.**
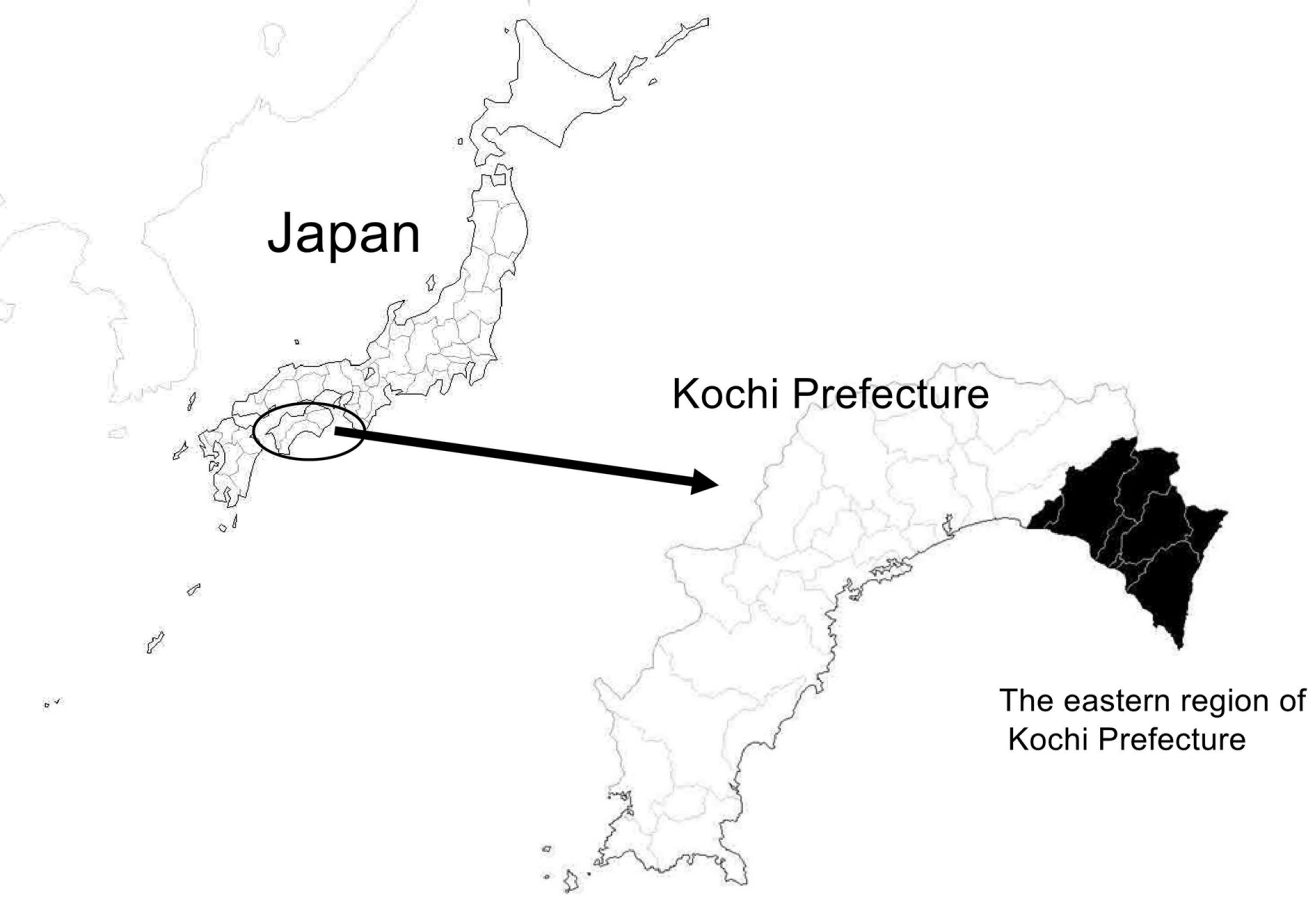
Location of the eastern region of Kochi Prefecture. The Kochi Prefecture is located in the southwestern part of Japan and has a mild and rainy climate. The eastern region of Kochi Prefecture is a blacktop area under the jurisdiction of the Aki Public Health Center of the Kochi Prefectural government.

Japanese spotted fever (JSF) is a tick-borne rickettsial disease that was first reported in Tokushima Prefecture in 1984 ^(1)^ and has been on the rise in recent years ^(2)^. The eastern region of Kochi Prefecture (Figure 1), under the jurisdiction of the Aki Public Health Center of the Kochi Prefectural Government (Aki HC: a division of Aki Welfare and Public Health Center), is considered a high-incidence area for JSF ^(3)^. It is an important region for the prevention of tick bite-related morbidity and the diagnosis and treatment of diseases with fever and rash, and JSF is a regional public health issue in this region.

I report on the incidence of JSF in Aki HC over 16 years from 2006 to 2021.

## Methods

In Japan, JSF is designated as a category IV infectious disease under the Infectious Disease Control Law, and all JSF cases are reported and registered in the National Epidemiological Surveillance of Infectious Disease (NESID) system operated by the National Institute of Infectious Diseases and the Ministry of Health, Labour and Welfare ^(4)^. I conducted a descriptive analysis of JSF cases registered in the NESID system from 2006 to 2021 using data from the Aki Public Health Center area.

The tabulated items included the number of cases (in the jurisdiction and nationwide), number of cases per 100,000 population (in the jurisdiction and nationwide), number of deaths, annual trends in the number of cases, number of cases per month, sex and age of patients, time from infection to disease onset, examination methods, and signs. Signs were tabulated for eschar at the site of the bite (eschar), stings, fever, rash, headache, abnormal liver function, and disseminated intravascular coagulation (DIC), which were mentioned on the notification form. The population was estimated on October 1 of each year, as calculated by the Kochi Prefecture and the Statistics Bureau of the Ministry of Internal Affairs and Communications (for census years, the Japanese population census was referred to).

## Results

The number of cases from 2006 to 2021 in the jurisdiction of the Aki HC was 70, with an incidence rate of 8.83 cases per 100,000 population. During the same period, the number of cases in Japan as a whole was 3,684, or 0.18 cases per 100,000 people, and the incidence rate was 48 times higher in the Aki HC area than in Japan. There was one fatality, and the fatality rate was 1.4%.

**Figure 2.**
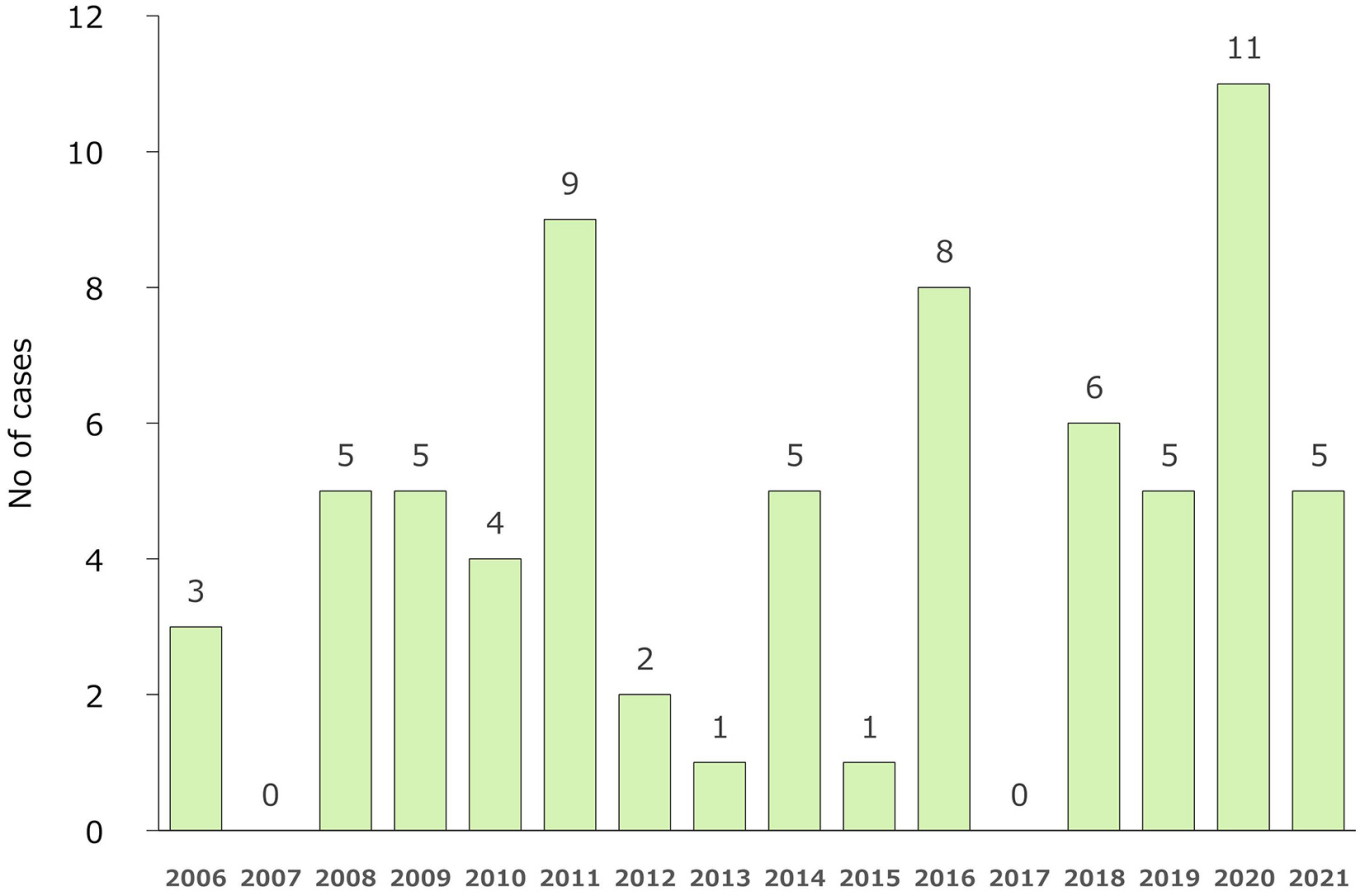
Annual trends in the number of cases of Japanese spotted fever reported. The number of cases varied from 0 to 11 per year.

**Figure 3.**
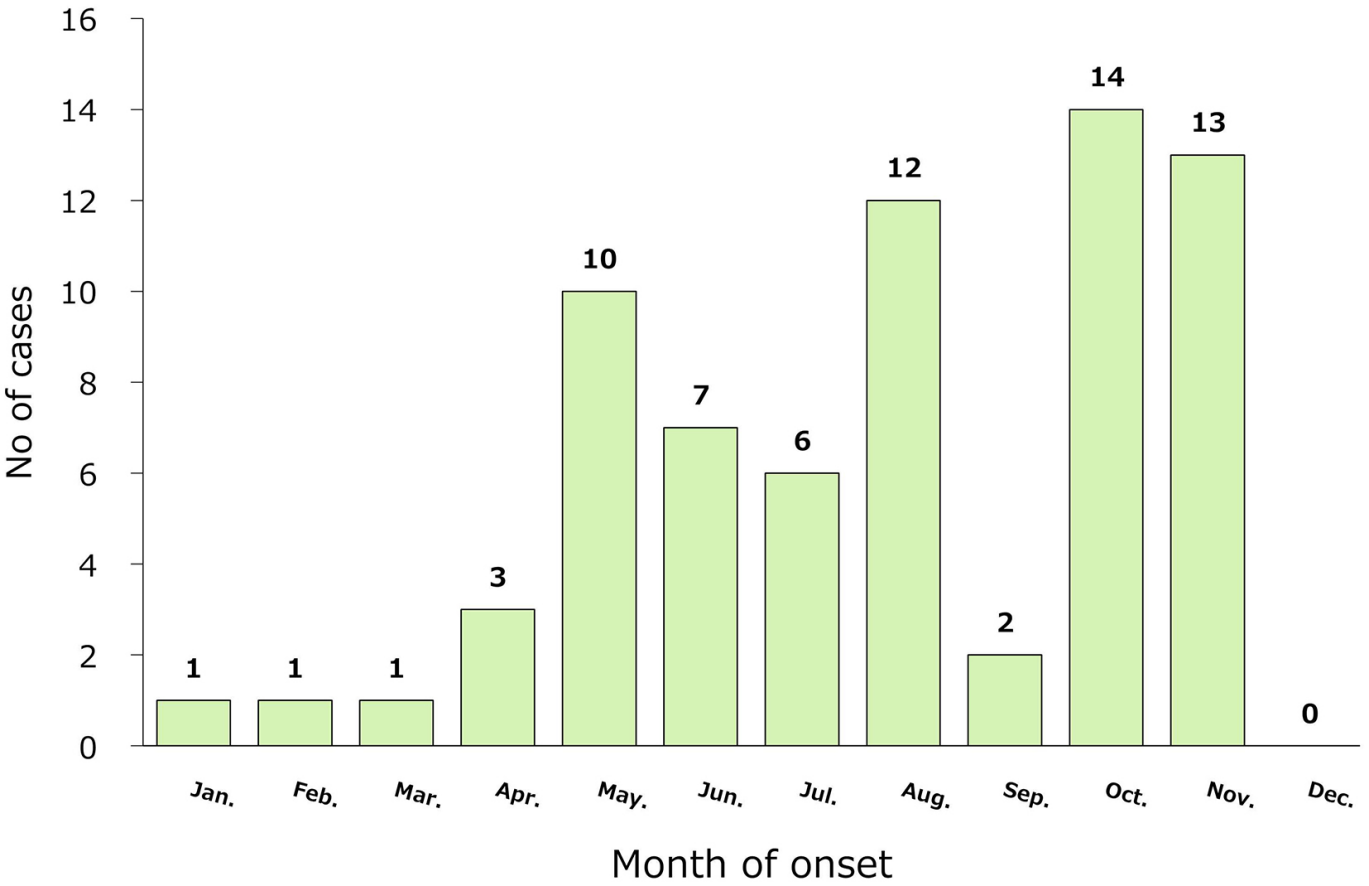
Number of cases of Japanese spotted fever by month. The number of cases per month was 14 in October, 13 in November, 12 in August, and 10 in May, with most cases occurring in autumn, early summer, and summer; however, cases also occurred in winter.

Figure 2 shows the annual trends, and the number of cases varied from 0 to 11 per year. There were 14 cases in October, 13 in November, 12 in August, and 10 in May, with most cases occurring in autumn, early summer, and summer; however, there were also cases in winter (Figure 3). Two (2.9%) patients were 50-59 years old, 16 (22.9%) were 60-69 years old, 21 (30.0%) were 70-79 years old, 28 (40.0%) were 80-89 years old, and 3 (4.3%) were ≥90 years old. Twenty-five (35.7%) patients were men and 45 (64.3%) were women. The incubation period before the onset of disease was 3.3 days in the 27 patients for whom the data of infection was estimated from epidemiological records. The methods of testing included serodiagnosis in 32 cases (no duplication with other tests), polymerase chain reaction (PCR) in 38 (including 28 cases of PCR using blood specimens and 20 cases of PCR using crusted specimens at the site of the bite, duplication), and virus isolation/identification in 2 (duplication with other tests). Since 2014, of 41 cases of JSF, 38 (92.7%) were laboratory-diagnosed by PCR.

Table 1 summarizes the clinical manifestations of the 70 cases. Eschar, fever, and rash were present in 62.9%, 98.6%, and 85.7% of the cases, respectively. Although these signs are known as the main symptoms of JSF, 57.1% (40/70) had all three signs (triad), 32.9% had two signs, 5.7% had eschar and fever, and 27.1% had rash and fever. Of the triad, only one sign was present in 10.0%, 1.4% of which had rash only, and 8.6% had fever only. Headache, liver function abnormalities, and DIC were present in 22.9%, 75.7%, and 40.0% of patients, respectively. Among the 30 patients who did not show any of the three signs, 76.7% had an abnormal liver function, 40.0% had DIC, and 6 (20.0%) had none of these complications. Of the six patients with only fever among the three signs, one had an abnormal liver function, two had DIC, and four (66.7%) had no complications.

**Table 1.** Signs of Japanese Spotted Fever Cases.

	No.		%	
Three signs (triad)				
Eschar at the site of the bite	44		62.9%	
Fever	69		98.6%	
Rash	60		85.7%	
				
All three signs were present	40		57.1%	
Two signs were present	23		32.9%	
Eschar and fever		4		5.7%
Rash and fever		19		27.1%
Eschar and rash		0		0.0%
One sign was present	7		10.0%	
Eschar only		0		0.0%
Fever only		1		1.4%
Rash only		6		8.6%
No signs were present	0		0.0%	
				
Other signs				
Headache		16		22.9%	
Liver function abnormalities		53		75.7%
DIC		28		40.0%

n = 70; DIC, disseminated intravascular coagulation

## Discussion

The eastern region of Kochi Prefecture (within the jurisdiction of Aki HC) had a very high incidence of JSF, which is considered an important disease that should be given a different diagnosis depending on the circumstances of febrile patients. The incidence of JSF increased from May to October and peaked from August to October; it coincided with the period of tick activity and is generally recognized as an infectious disease that occurs from spring to fall ^(5)^. However, JSF can occur even in the relatively cold season if there is contact with ticks, such as during fieldwork. In my sample, there were more female patients than males, similar to a recent report ^(6)^. The older age of the patients may reflect the sex ratio in the older population.

The three signs of JSF―fever, rash, and eschar―were present in 98.6%, 85.7%, and 62.9% of the cases, respectively. However, 57.1% of the cases presented all three signs. These results suggest that JSF should be suspected in JSF endemic areas, even in the absence of eschar. According to the recent reports from hospitals in Japan, Sakabe et al. have found fever and rash in 99.6% and eschar in 61.9% of JSF patients, indicating a similar prevalence to that in the present study ^(6)^. Contrarily, Sando et al. have reported that rash is prevalent in 100% and eschar in 89% of JSF patients, based on the physician’s examination at the first visit; however, 60% of the patients themselves are aware of the rash, and only 4% are aware of the eschar ^(7)^. Although rashes or eschars may be difficult for the patient to recognize, the lesions should be carefully examined visually by a physician. Four patients with only fever and no liver function abnormalities and DIC were reported, whereas previous studies have reported that patients with JSF had higher C-reactive protein levels ^(1), (8)^, low platelet counts ^(1), (2)^, and abnormal urine findings ^(1)^. These findings were not required to be reported in the incidence report, and these abnormal findings may coexist. As the NESID system has these limitations, future analyses, including case studies, should be considered.

In JSF endemic areas, JSF should be considered as a cause of fever and rash, regardless of the season. If JSF is suspected, the immediate administration of antimicrobials (tetracyclines) is recommended without waiting for a laboratory diagnosis ^(2), (9)^ Additionally, in all seasons, including the cold season, to avoid tick bites, local residents are required to wear protective clothing and use tick repellents during fieldwork.

## Article Information

### Conflicts of Interest

None

### Acknowledgement

I am grateful to the notifying clinicians for providing information on rickettsiosis notifications.

### Author Contributions

The author collected all the data used in this research and wrote the manuscript.

### Approval by Institutional Review Board (IRB)

Ethical approval was not required because this study was conducted for public health purposes using legally notifiable surveillance data. Identifiable information is not reported here.
